# Transcriptional and posttranscriptional regulation of *Bacillus* sp. CDB3 arsenic-resistance operon *ars1*

**DOI:** 10.7717/peerj.1230

**Published:** 2015-09-03

**Authors:** Xuefei Yu, Wei Zheng, Somanath Bhat, J. Andrew Aquilina, Ren Zhang

**Affiliations:** School of Biological Sciences, University of Wollongong, Wollongong, NSW, Australia; 1 Current affiliation: Research Center on Life Sciences and Environmental Sciences, Harbin University of Commerce, Harbin, China; 2 Current affiliation: National Measurement Institute of Australia, Lindfield, NSW, Australia

**Keywords:** Arsenic resistance, Operon expression, RNA degradation, Transcription attenuation

## Abstract

*Bacillus* sp. CDB3 possesses a novel eight-gene *ars* cluster (*ars1, arsRYCDATorf7orf8*) with some unusual features in regard to expression regulation. This study demonstrated that the cluster is a single operon but can also produce a short three-gene *arsRYC* transcript. A hairpin structure formed by internal inverted repeats between *arsC* and *arsD* was shown to diminish the expression of the full operon, thereby probably acting as a transcription attenuator. A degradation product of the *arsRYC* transcript was also identified. Electrophoretic mobility shift analysis demonstrated that ArsR interacts with the *ars1* promoter forming a protein-DNA complex that could be impaired by arsenite. However, no interaction was detected between ArsD and the *ars1* promoter, suggesting that the CDB3 ArsD protein may not play a regulatory role. Compared to other *ars* gene clusters, regulation of the *Bacillus* sp. CDB3 *ars1* operon is more complex. It represents another example of specific mRNA degradation in the transporter gene region and possibly the first case of attenuator-mediated regulation of *ars* operons.

## Introduction

Arsenic compounds are widely dispersed in the environment, which poses a threat to all living organisms. Many bacteria have evolved arsenic resistance (*ars*) operons located on either chromosomes or plasmids, which encode a specific detoxification pathway for arsenic extrusion (Rosen, 2002; Kruger, Bertin & Heipieper, 2013).

The transcription pattern of a common three-gene *ars* operon (*arsRBC*) is relatively simple: intracellular arsenite binds to the repressor (ArsR), inducing a conformational change of the protein and dissociation from the promoter, thereby allowing expression to proceed (Shi, Wu & Rosen, 1994). The transcriptional regulation of a common five-gene *ars* operon (*arsRBCDA*) has also been well characterised and a model was proposed (Chen & Rosen, 1997). The initiation of operon transcription is the same as *arsRBC*; however, when the membrane transporter ArsB protein is largely expressed, it may become toxic to host bacterial cells. Therefore, transcription of the operon can be repressed via the second repressor ArsD binding to the regulatory region, to which ArsR binds (Bruhn et al., 1996), even though ArsR and ArsD share no significant sequence similarity (Chen & Rosen, 1997). With the increasing concentration of arsenite, ArsD tends to dissociate from operator region, re-activating transcription of *ars* operon. This mode employs a secondary repressor to guard the upper level of gene expression, but still only involves single transcript. Apart from these two well characterised regulatory mechanisms, other transcriptional modes have also emerged along with the discoveries of more *ars* operons. For example, the basic repressor ArsR is not always operon born. The *arsBHC* operon of *Synechocystis* sp. Strain PCC6803 missing transcriptional repressor gene has been proved to be mediated by an ArsR protein that is encoded away from the operon (López-Maury, Florencio & Reyes, 2003). Similarly, the *Ochrobactrum tritici* SCII24 *arsR1C1YC2H* operon is also regulated by another ArsR, which is constitutively expressed and resides outside the operon in opposite direction (Branco, Chung & Morais, 2008). Moreover, some bidirectional *ars* operons have been found mediated by single ArsR but expression levels of two divergent transcripts appear different in response to arsenic stress changes, such as the *ars* operons from *Acidithiobacillus ferrooxidans* (Butcher & Rawlings, 2002), *Corynebacterium glutamicum* ATCC13032 (Ordóñez et al., 2005), *Microbacterium* sp. Strain A33 (Achour-Rokbani et al., 2010) and pR478 of *Serratia marsescens* (Ryan & Colleran, 2002).

*Bacillus* sp. CDB3 is a highly arsenic-resistant bacterium, containing two *ars* clusters (Bhat et al., 2011). The *ars2* is similar to the four-gene operon *arsRorf2YC* (*Y* represents *yqcL*) from the skin element of *B. subtilis* (Sato & Kobayashi, 1998), while *ars1* is one of the longest *ars* clusters characterised to date, bearing eight genes (*arsRYCDATorf7orf8*) of which two are novel (Bhat et al., 2011). The *orf7* gene encodes a putative HesB-like protein and *orf8* a putative dual-specificity protein phosphatase (DSP). Assessed in *E. coli* host, *orf8* exhibited a significant resistance to arsenate but it could not complement the function-loss of *arsC* gene, indicating its role not as an arsenate reductase (Zheng et al., 2013). In addition to these new genes, *ars1* also displays features consistent with a novel regulation mechanism (Bhat et al., 2011). (1) Arrangement of the five genes (*arsRYCDA*) differs from the common five-gene *ars* operon (*arsRDABC*) in *E. coli*; (2) two inverted repeats exist between *arsC* and *arsD* potentially forming a hairpin structure, which may play a role in transcriptional regulation; and (3) the ArsD protein sequence lacks the C-terminal vicinal cysteine pairs which *E. coli* ArsD bears, bringing into question its role as a repressor regulator. In this study, we investigated the transcriptional pattern of CDB3 *ars1* under varying arsenic stresses and assessed the function of potential regulatory factors.

## Materials and Methods

### Bacterial strains, plasmids and DNA primers

The bacterial strains, plasmids and primers used in this study are listed in Table 1. LB media with appropriate antibiotics or arsenic compounds were used to culture *Bacillus* sp. CDB3 at 30 °C or *E. coli* strains at 37 °C, respectively.

**Table 1:** Bacterial strains, plasmids and DNA primers used in this study.

Strains, plasmids and primers	Description	Source
**Strains**		
*Escherichia coli*		
JM109	*rec*A1, *end*A1, *gyr*A96, *thi*, *hsd*R17 (rk^−^, mk^+^), *sup*E44, *rel*A1, Δ(*lac*-*pro*AB), [F’, *tra*D36, proAB, *lac*IqZΔ M15]	Promega (USA)
AW3110	K-12 F-IN (rrnD-rrnE) *ars*::*cam* (Cm^*r*^, the chromosomal *arsRBC* deleted)	Gift of Dr BP Rosen (Carlin et al., 1995)
M15 (pREP4)	*na*I^*s*^, *str^s^*, *rif^s^*, *thi*^−^, *lac*^−^, *ara*^+^, *gal*^+^, *mtl*^−^, F^−^, *rec*A^−^, *uvr*^+^, *lon*^+^	Promega (USA)
*Bacillus* sp. CDB3	Isolated from cattle dip sites	Chopra et al. (2007)
**Plasmids**		
pAR27	p*RYCDATorf7orf8*, CDB3 *ars1*cloned in pGEM7Zf(+)	Bhat et al. (2011)
pQE30	N-His_6_ expression vector, *P*_*T*5_/*O_lac_*, ColEI ori, Ap^*r*^	Qiagen (Germany)
pQENR	pQE30/ArsR expression plasmid	This study
pQEND	pQE30/ArsD expression plasmid	This study
pAR27HP^Δ^	Mutant of pAR27 in the inverted repeat region between *C* and *D*	This study
**Primers (5’-3’)**		
Probe 1F	CCATAACTTGCACCCAC	
Probe 1R	TTAACAAGAGTGTCACAG	
Probe 2F	GTCTGGAGCTTGCACAG	
Probe 2R	GGACTTCAGATTGTGATCGTC	
Probe 3F	TCTGCAATGGAAGAAGC	
Probe 3R	GTCTAGACTGTTTCTGTGACATC	
ProbeR-F	TTAACAAGAGTGTCACAG	
ProbeR-R	CTCTGTTTGGTCTGGTAC	
ProbeC-F	TTACCATTGACCGTGAC	
ProbeC-R	TCTCCTGTTTCAGCGAAT	
RT-DF	CCGAGTGTTGACCCAGAGTT	
RT-DR	CATCTGGTTCACTTGCCAAA	
pQENR-F^a^	(*Bam*HI)CCA**GGATCC**ATGGAAAAAACGGTTATAG	
pQENR-R^a^	(*Hin*dIII)TAT**AAGCTT**TTAACAACCACACCGGAG	
pQEND-F^a^	(*Bam*HI)CCA**GGATCC**ATGAAGAAGATAGAAATTTTTGATCCTG	
pQEND-R^a^	(*Hin*dIII)TAT**AAGCTT**CTATTTTTTCACGTTTAACTTTAAACGTACGACTGG	
ProR-F	TTCAGTTGAATATATAAGCG	
ProR-R	TATCTAGAGCCATATCTATACCTCCTT	
ProN-F	TGAGACTGCTATGAAAG	
ProN-R	GTCTAGACTGTTTCTGTGACATC	
PED-R^b^	ACATTAATACAGGTCCAATAATCCAGTTT	

**Notes.**

a The added restriction enzyme sites are shown in bold type.

b Labelled with 6FAM.

### Northern analysis

Cell pellets of appropriately treated cultures at mid-exponential growth phase were suspended in NETS buffer (0.1 M NaCl, 10 mM Tris-HCl pH 8.0, 1 mM EDTA, 1% SDS) followed by phenol-chloroform extraction. Total RNA in the aqueous phase was collected by ethanol precipitation and then redissolved in RNase-free water. Probes were produced from pAR27 by PCR amplification using a DIG PCR labelling kit (Roche, Penzberg, Germany) following manufacturer’s protocol. For northern blotting, 10 µg of total RNA was loaded per lane and run in 1.8% (w/v) agarose denaturing formaldehyde gels in MOPS buffer (20 mM MOPS, 5 mM sodium acetate, 10 mM EDTA, pH 7.0) before being transferred onto nylon membranes (Hybond™-N+; Amersham plc, Amersham, UK). Prehybridization, hybridization, washes and film (Kodak, Rochester, New York, USA) image development were conducted as described in the product manuals.

### Real-time qPCR and primer extension assay

Total RNA samples isolated above were treated with RQ1 RNase-free DNase (Promega, Madison, Wisconsin, USA) before cDNA synthesis using a Transcriptor First Strand cDNA Synthesis kit from Roche following manufacturer’s instruction. Real-time qPCR to quantify *arsD* cDNA with primer pair RT-DF/RT-DR was carried out using a SensiFAST™ SYBR No-ROX kit (BioLine, Taunton, Massachusetts, USA) on a LightCycler@480 detection system (Roche, Penzberg, Germany). The PCR conditions were: one cycle of 95 °C for 5 min; 45 cycles of 95 °C for 10 s, 60 °C for 20 s and 72 °C for 10 s. Each run was followed by a melt analysis comprising a 2.2 °C/s ramp rate from 95 °C to 65 °C. The cDNA was quantified using a Nanodrop^®^ ND-1000 spectrophotometer.

Primer PED-R labelled with 5’-end 5-6-carboxyfluorescein (6FAM) was used in reverse transcription before the resultant cDNA products were examined using an ABI^®^ 3130xl Genetic Analyzer (Applied Biosystem, Carlsbad, California, USA) with GeneScan™-500 LIZ^®^ internal size standards (Applied Biosystem, Carlsbad, California, USA) and analysed using GeneMapper^®^ software (Version 4.0).

### Construction of hairpin mutant strain

To eliminate the inverted repeats between *arsC* and *arsD*, a mutant 638-bp DNA was synthesised (GeneArt, Bavaria, Germany) to replace the original *Kpn*I-*Rsr*II fragment in pAR27. The resultant plasmid pAR27HP^Δ^ was introduced into *E. coli* AW3110 for RNA assays.

### Electrophoretic mobility shift assay

The coding regions of *arsR* and *arsD* were amplified from pAR27 and cloned into pQE30 (Qiagen, Hilden, Germany) at *Bam*HI and *Hind*III sites resulting in His-tag recombinant vectors pQENR and pQEND, respectively. Upon sequence verification, the two constructs were transformed into *E. coli* strain M15 followed by induction at 37 °C for 4 h using 0.5 mM isopropyl *β*-D-1-thiogalactopyranoside (IPTG). Cell pellets were lysed by French press (SLM Aminco, Rochester, New York, USA) at 20,000 psi. The target recombinant proteins were isolated and purified from the filtered lysates using HisTrap™ HP, HiLoad16/60 Superdex75 and Vivaspin 10 kDa MWCO columns (GE healthcare, Wauwatosa, Wisconsin, USA) and then stored in small aliquots at −80 °C before use.

A 167-bp fragment covering the promoter region of *ars1* was PCR amplified using primer pair ProR-F/ProR-R from plasmid pAR27 and named *proR*. Another 132-bp fragment within *orf7* was also amplified using ProN-F/ProN-R as a non-specific DNA control named *proN*. After purification by Sephadex G50 spin column chromatography (Sambrook, Fritsch & Maniatis, 1989), the PCR products were incubated with the above purified His-ArsR or His-ArsD proteins in binding buffer (10 mM Tris-HCl pH 7.6, 80 mM KCl, 0.2 mM EDTA, 0.2 mM dithiothreitol, 10% glycerol and 0.75 µM BSA; 30 µL) on ice for 30 min. The samples were then loaded onto a 6% non-denaturing acrylamide gel and run at 120 V for 40 min in TBE buffer (pH 8.3) before staining with ethidium bromide.

## Results and Discussion

### Transcript analysis of CDB3 *ars1*

**Figure 1:**
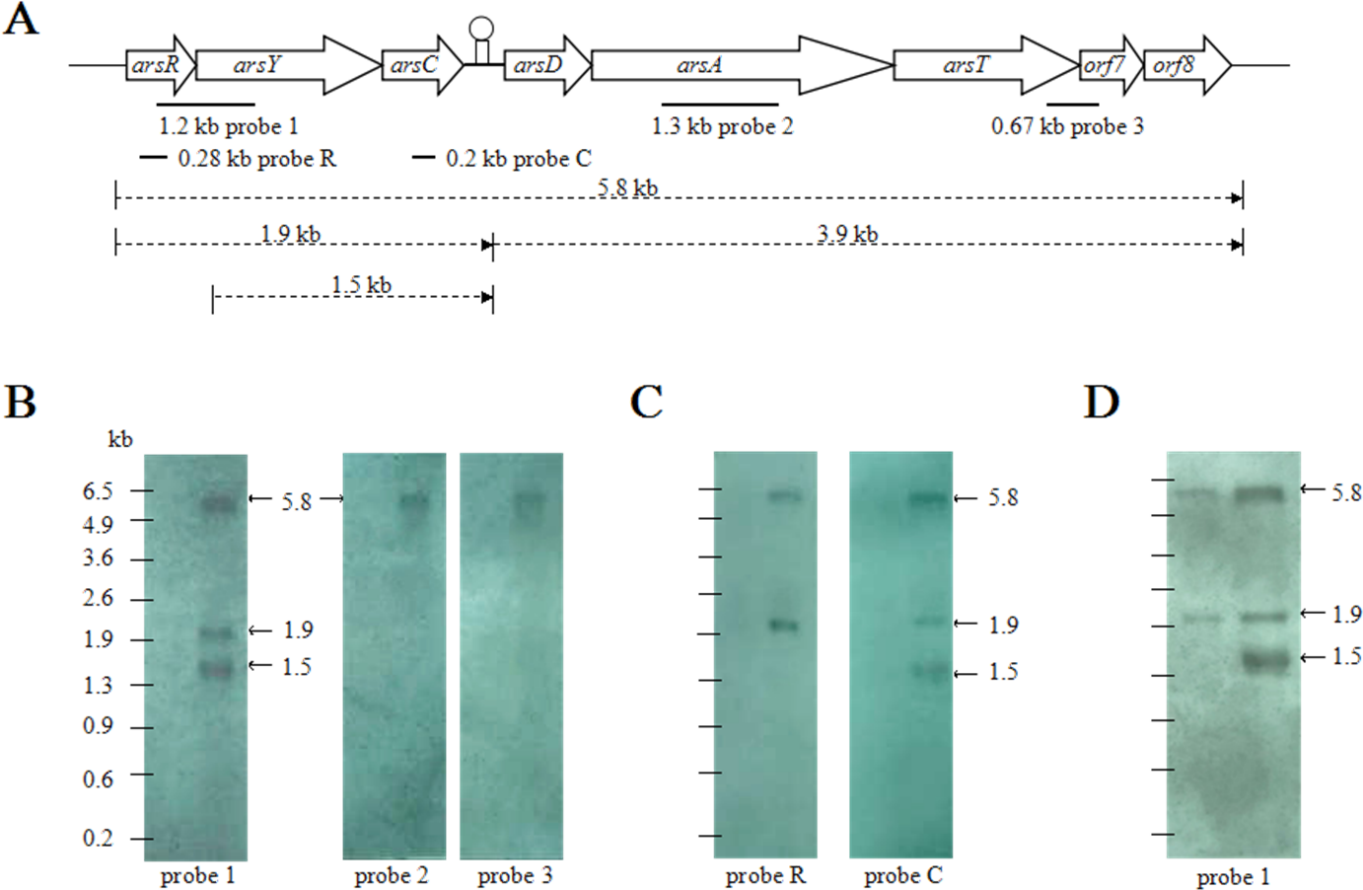
Northern blotting analyses of CDB3 *ars1* expression. (A) A diagram of CDB3 *ars1* labelled with locations of probes used and possible transcript sizes (kb). (B) Images of northern blot ting using probes 1, 2 and 3. On each blot the left lane is untreated control and right is arsenite-treated at 2 mM/10 min. (C) Images of northern blot ting using probes R and C. On each blot the left lane is untreated control and right is arsenite-treated at 0.5 mM/5 min. (D) Images of northern blot ting using probe 1 of samples treated with 4 mM arsenite for 3 min (left) and 30 min (right).

To examine the transcription pattern of CDB3 *ars1*, total RNA extracts from arsenite-stressed and control bacterial cells were first analyzed by northern blot analysis using three probes, covering different regions of the cluster (Fig. 1A). While there was no hybridization signal detected in the control RNA sample, arsenite treatment induced the expression of *ars1* and a 5.8-kb transcript corresponding to the full cluster length was detected by all three probes (Fig. 1B). This suggests that *arsRYCDATorf7orf8* can be transcribed as a single polycistronic mRNA and that there is only one promoter at the 5’-end of the cluster. In addition, probe 1 (covering region *RY*), but not probes 2 and 3 (covering *A* and *Torf7,* respectively), detected two other RNA fragments of approximately 1.9 kb and 1.5 kb. A further northern blot assay (Fig. 1C) demonstrated that probes R and C (covering internal fragments of *arsR* and *arsC*, respectively) can both hybridize to the 1.9-kb RNA band, corresponding to the length of *arsRYC* but the 1.5-kb RNA band was only detected when probe C was applied. It was also observed that the amount of 1.5-kb RNA increased with treatment time, which is evident in the 4 mM arsenite samples: at 3 min the 1.5-kb RNA was not detected but was remarkably abundant after 30 min (Fig. 1D). This suggests that the first three genes, *arsRYC*, can also be transcribed as a polycistronic unit during *ars1* expression and that the inverted repeat between *arsC* and *arsD* may function as a read-through transcriptional attenuator. Additionally, the 1.5-kb RNA band was found to lack the *arsR* gene compared with the 1.9-kb RNA fragment. Thus, it was assumed to be a possible differential mRNA degradation product spanning the *YC* gene region.

### Function of the internal hairpin structure

**Figure 2:**
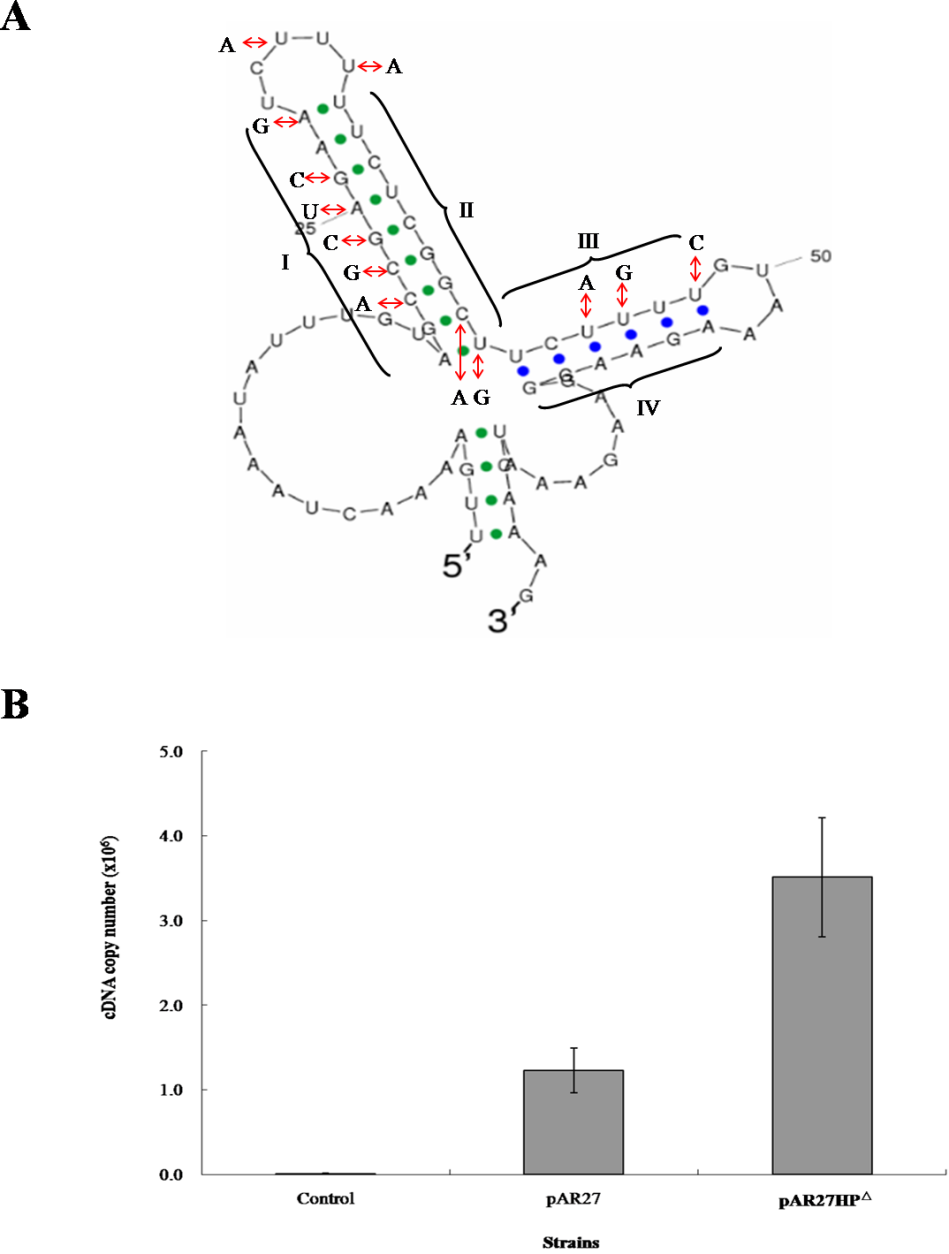
mRNA secondary structure and long-transcript levels. (A) Predicted mRNA secondary structure of the *C–D* intergenic region in CDB3 *ars1* and altered nucleotides to abolish the hairpin structure. The four regions of the two stem-loops are indicated by symbols I, II, III and IV. (B) Expression levels of the long transcript in untreated (control) and arsenite-treated *E. coli* AW3110 strains harbouring pAR27 or pAR27HP^Δ^. The arsenite treatment was at 0.5 mM for 5 min and qPCR was carried out using RT-DF/RT-DR primer pairs. Each data point corresponds to average copies of ArsD cDNA (copies/µL) and the error bars indicate standard deviation of three independent measurements.

To examine the role of inverted repeats between *arsC* and *arsD*, which can form a hairpin structure, the intergenic DNA sequence was altered by mutagenesis to eliminate the repeats (Fig. 2A). A transcription study was carried out in *E. coli* AW3110, which was shown to produce the two RNA transcripts when transformed by CDB3 *ars1* (data not shown). RNA samples isolated from AW3110 strains harbouring pAR27 (*ars1*) or pAR27HP^Δ^ (hairpin knockout mutant) with or without arsenite treatment were reverse transcribed and analysed using qPCR assay. The results showed that in the mutant strain, expression levels of the long eight-gene transcript increased more than one fold over that in the control strain (Fig. 2B), suggesting that the hairpin structure does function as a transcriptional terminator which can be read through under some conditions.

Transcription attenuation has been recognised as a strategy in bacteria to modulate expression of operons through transcription pausing or termination, often in response to extracellular environmental changes. The region between *arsC* and *arsD* was predicted to form two stem-loops after transcription due to the presence of inverted repeat sequences. The first one (23-bp) displays features of a Rho-independent terminator, where GC is rich in the repeat followed by concentrated U nucleotides (Fig. 2A) which is common in *Bacillus* but also in *E. coli* (Naville & Gautheret, 2010). Terminator read-through via anti-termination has been demonstrated as a mechanism of transcriptional attenuation (Naville & Gautheret, 2010). In the case of CDB3 *ars1,* the predicted internal terminator structure may be tackled by an anti-terminator protein, which would stabilise the anti-terminator structure allowing production of the long eight-gene transcript. Alternatively, the anti-termination read-through may be via a riboswitch mechanism (Naville & Gautheret, 2010). Hence, the formation of a hairpin structure to terminate transcription after *arsC* in CDB3 *ars1* RNA might be interfered with by some small molecule(s). This type of anti-termination usually involves an anti-terminator (Naville & Gautheret, 2010); however, our sequence analysis did not identify an anti-terminator structure in the RNA region. One other possibility is a weak termination without actual regulation by an anti-terminator or protein. Such a terminator has been found in the *E. coli lac* operon between *lacZ* and *lacY*. However, switch control function of the terminator was only speculated under some specific physiological circumstances with no experimental evidence obtained (Murakawa et al., 1991). Since ArsY and ArsC produced from the short transcript *arsRYC* can provide quite significant resistance, along with the downstream gene products conferring additional tolerance (Zheng et al., 2013), it is reasonable to assume that employing a switch to regulate the two alternative transcription processes would benefit the host bacteria in terms of energy consumption. Further investigation is warranted. A database search also revealed that a number of *Bacillus* strains, such as *Bacillus cereus* ATCC10987 and *Bacillus thuringiensis* BM1904.1B171, harbor a similar seven-gene operon (*arsRYCDAOrf7,8*, only lack of *arsT*). They all contain a hairpin sequence in the intergenic region between *arsC* and *D* so assumingly have the similar transcription pattern as CDB3 *ars1*. Such internal terminator-like structures my also exist in other types of *ars* operons.

### Site specific cleavage of *arsY* RNA

**Figure 3:**
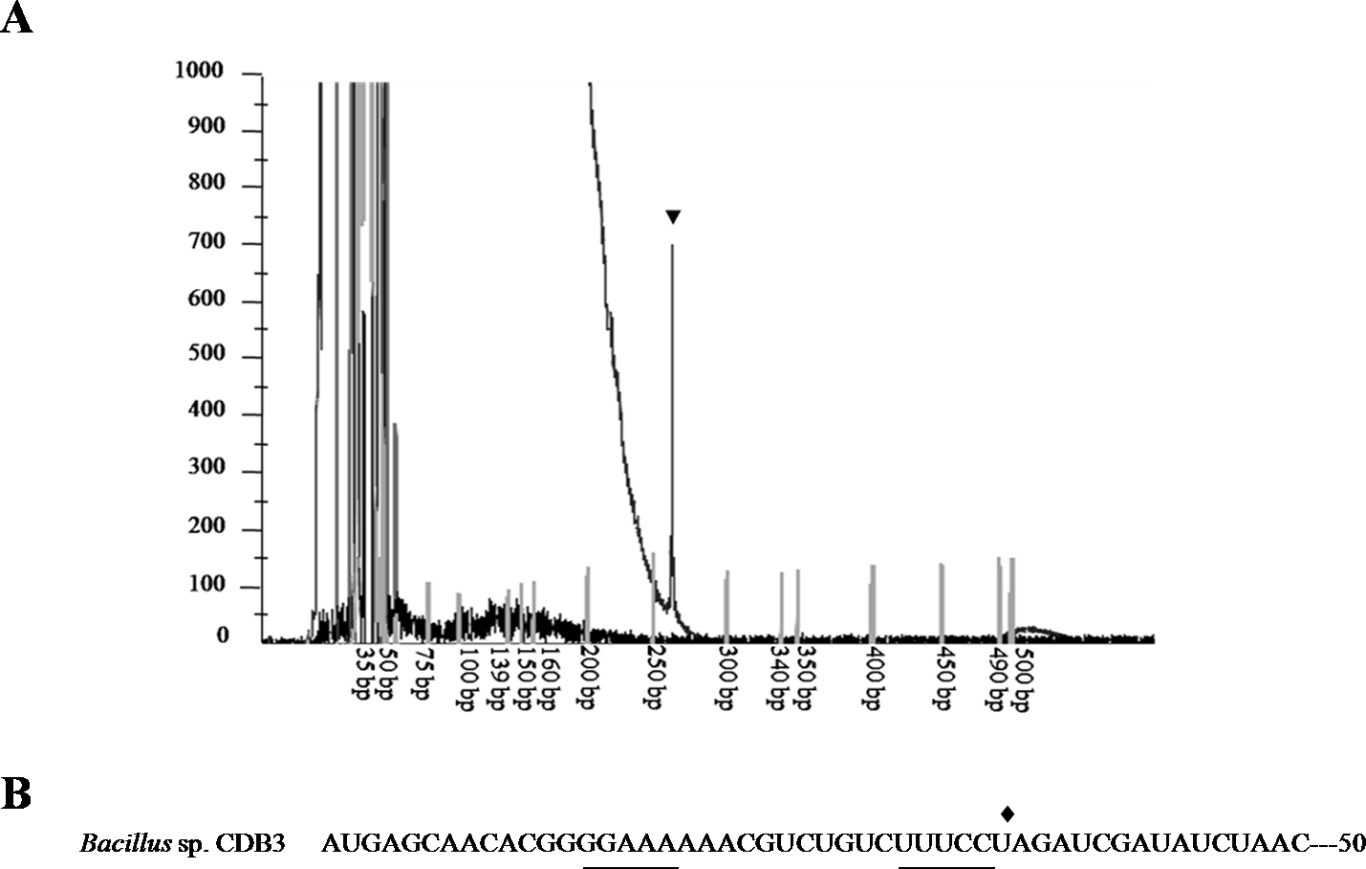
Mapping of RNA degradation product. (A) Electropherogram showing the result of primer extension assay. The extension product is indicated by arrowhead and size standards are labelled below. (B) The first 50 nucleotides sequence of CDB3 *arsY* RNA coding region indicating an inverted repeat (underlined) and 5’-end of 1.5-kb RNA (pointed by diamond).

To confirm the identity of the 1.5-kb RNA, a primer extension assay was conducted to map the 5’-end. Fluorescent labeled primer PED-R (29-bp) was annealed from 295 bp downstream of the *arsY* coding region. The electropherogram of extension products showed a predominant peak corresponding to a 261-bp cDNA fragment (Fig. 3A), demonstrating that the 5’-end of 1.5-kb RNA was the 35th bp of *arsY*. This nucleotide (uridine) is immediately downstream of an inverted repeat region in *Y* RNA sequence (Fig. 3B). This further supports the assumption that the smaller RNA resulted from degradation.

Differential rate of RNA degradation is a post-transcriptional regulation strategy and has been studied in different living organisms including bacteria (Houseley & Tollervey, 2009). A previous report on the *E. coli* plasmid R773 *arsRDABC* operon found rapid degradation of ArsB messenger and proposed that two RNA hairpins, one in the intergenic region before *arsB* and the other in the 5’ end of *arsB*, are responsible (Owolabi & Rosen, 1990). Cai & DuBow (1996) also communicated a similar case for the *E. coli* chromosomal *arsRBC* operon. Our finding that the CDB3 *arsY* RNA also cleaved in the 5’ region adjacent to a hairpin supports the notion that RNA secondary structure is important for degradation (Owolabi & Rosen, 1990). Compared with *E. coli* RNA, *Bacillus* sp. CDB3 RNA has only one hairpin located between the 5th and 12th codons of *arsY* which is equivalent to the second hairpin present in the *E. coli* counterpart (from 3rd codon of *arsB* RNA) (Owolabi & Rosen, 1990), with a free energy of −7.0 kcal/mol. This secondary RNA structure may limit the translation of ArsY as suggested for the *E. coli* hairpin on ArsB translation (Owolabi & Rosen, 1990). However, in the absence of a second upstream hairpin (as found in *E. coli* mRNA), it also assumes a main role in the specific degradation. The 5’ end of a degradation product has been identified as U^35^ which is straight after the hairpin, not before, suggesting a cleavage point between C^34^ and U^35^ (Fig. 3). Although only the prominent band of *arsYC* RNA fragment (1.5 bp) was identified, it is reasonable to assume that this specific cleavage also took place in the full operon transcript, which was not observed due to co-migration of the cleaved (∼5.4 kb) and intact RNA (∼5.8 kb) on our RNA gels (Fig. 1). The upstream part of transcript after cleavage (∼400 bp: *arsR* plus 34 bp of *Y*) was not detectable by northern analysis, indicating that it had been rapidly degraded. Unlike the first hairpin in *E. coli* R773 (Owolabi & Rosen, 1990), the 5-bp repeat stem at the 3’ end of the degradation product is probably not strong enough to act as a decay terminator. Nevertheless, our results demonstrate another case of specific degradation for *ars* polycistronic RNA in the transporter gene regions, suggestive of differential rate of RNA degradation, which helps prevent toxic levels of hydrophobic transporter proteins (Owolabi & Rosen, 1990). This regulatory pathway controlling intracellular protein concentration at the expression level may be common to a variety of bacteria.

### Confirmation of ArsR as a repressor of *ars1*

**Figure 4:**
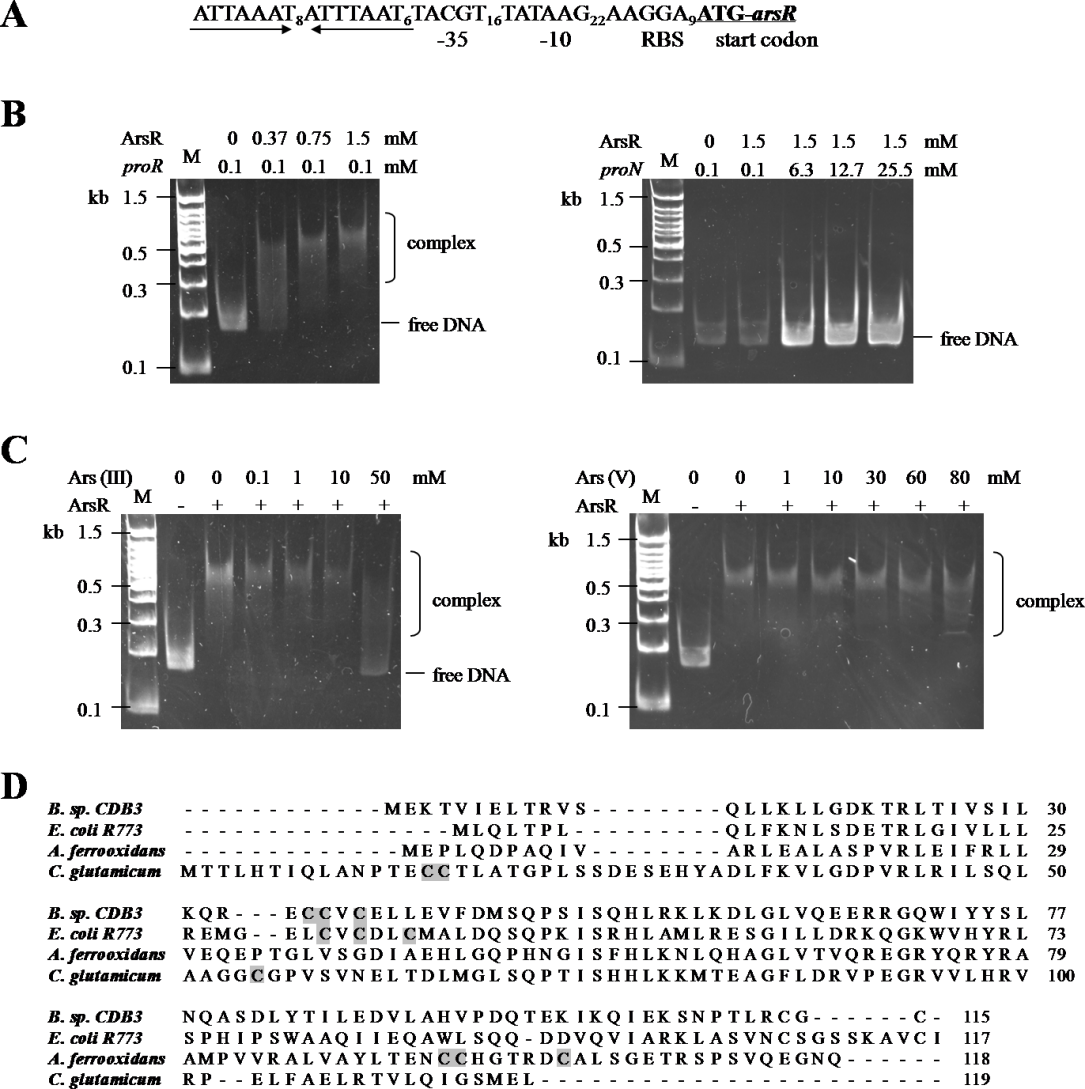
Binding motifs and mobility shift assays of ArsR. (A) Putative promoter region in *ars1*. The inverted repeat is marked by inverted arrows lines . The putative ribosomal binding site (RBS), −35 and −10 boxes and start codon of *arsR* are indicated. (B) Examination of ArsR binding. Left: ArsR binding with *proR*. The amounts of DNA fragment and protein used were indicated above the panel. Right: ArsR binding with *proN*. ArsR was incubated with *proN* at equal and much higher concentrations than that used for *proR*. (C) Effect of arsenite (Left) and arsenate (Right) on ArsR-DNA complex. Each reaction contains 0 .1 mM *proR* and 0.75 mM of ArsR (+) or 0 mM of ArsR (−).The arsenic concentrations in each reaction are shown on top of panel. Lane M is a 100 bp DNA ladder with representative sizes indicated. (D) Multiple ArsR sequences alignment . Representative homologues (accession numbers in parentheses) are from *E. coli* R773 (P15905), *Bacillus* sp. CDB3 (AF178758), *Acidithiobacillus ferrooxidans* (AAF69241) and *Corynebacterium glutamicum* (YP_225794.1). The identified or predicted metalloid binding cysteines are highlighted in shadow.

The CDB3 ArsR protein is homologous to the *ars* operon regulator of *Bacillus subtilis* Skin element with 89% similarity (Bhat et al., 2011), suggesting a potential repressor function in *Bacillus* sp. CDB3. Sequence analysis of the *ars1* promoter region identified a 22-bp inverted repeat (Fig. 4A), probably acting as repressor binding site. With the recombinant CDB3 ArsR protein produced and purified from *E. coli*, an electrophoretic mobility shift assay (EMSA) demonstrated that the ArsR protein retarded the mobility of a 167-bp DNA fragment (*proR*) containing the promoter region of *ars1* by forming a DNA-protein complex (Fig. 4B left). At a fixed concentration (0.1 mM) of DNA, the binding was enhanced along with increasing concentrations (0.37–1.5 mM) of ArsR. However, there was no interaction observed when the protein was incubated with a non-specific DNA fragment (*proN*) even at 25.5 mM (255-fold concentration as that used for *proR*) (Fig. 4B right), which confirmed the binding specificity between ArsR and the *ars1* promoter. Since the transcription of *ars1* can be induced by arsenic, interruption of the binding by arsenic compounds was assessed. As shown in Fig. 4C (left), with the concentration of arsenite increased the protein-DNA complex was gradually disrupted and a complete separation occurred at 50 mM. However, arsenate did not affect the ArsR-DNA complex even at a concentration of 80 mM (Fig. 4C right). Therefore, arsenite, but not arsenate, is capable of binding CDB3 ArsR, leading to dissociation from the *ars1* promoter.

ArsR is a member of ArsR/SmtB family identified from an extensive range of microbes, responding to transition metals, heavy metals and metalloids (Pennella & Giedroc, 2005). Investigations of this transcriptional repressor suggest that they share a winged helix protein as ancestor but their inducer binding sites are diverse and appear to evolve independently (Osman & Cavet, 2010). The *Bacillus* CDB3 ArsR possesses three cysteine residues: Cys^35^, Cys^36^ and Cys^38^ in the ELCVCDL binding motif, which vary in positions from other known ArsRs (Fig. 4D). The typical ELCVCDL motif of metal(oid)-binding site consists of Cys^32^, Cys^34^ and Cys^37^ adjacent to a DNA binding domain, represented by *E. coli* R773 ArsR (Shi, Wu & Rosen, 1994). By contrast, the metalloid binding site of ArsR in *Acidithiobacillus ferrooxidans* is composed of Cys^95^, Cys^96^ and Cys^102^ contiguous to the C-terminal dimer interface (Qin et al., 2007) and that in *Corynebacterium glutamicum* consists of Cys^15^, Cys^16^ and Cys^55^ (Ordóñez et al., 2008). The three sulfur thiolates have been proposed to form a pyramidal cage to specifically bind trivalent arsenite which is three-coordinate (Shi et al., 1996).

### ArsD may not function as a repressor of *ars1*

**Figure 5:**
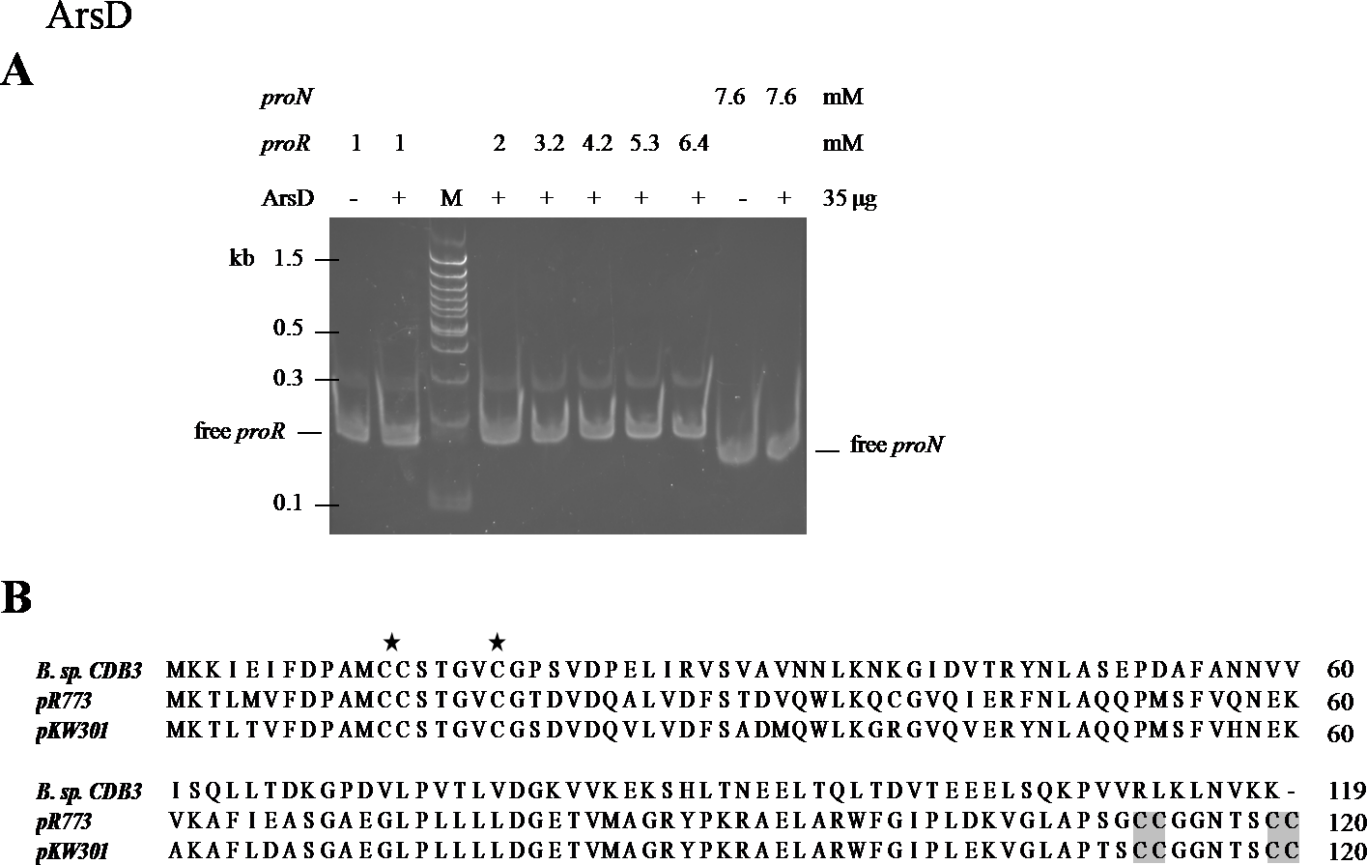
Mobility shift assay of ArsD. (A) EMSA of ArsD binding to *proR* and *proN*. Indicated amount of each DNA fragment binds with (+) or without (−) ArsD. M is a 100 bp DNA ladder with representative sizes indicated. (B) Sequence alignment of ArsDs from *Bacillus* sp. CDB3 (AAD51848.1), *E. coli* pR773 (AAA93060) and *Acidiphilium multivorum* pKW301 (BAA24821). Accession numbers are in parentheses. The stars indicate conserved Cys in all three sequences and shadow indicates those not present in *Bacillus* sp. CDB3 ArsD.

An EMSA was also carried out using recombinant CDB3 ArsD protein to test its ability to bind DNA. Such a binding affinity was not detected so the results did not show a mobility shift of *proR* DNA after reaction with the ArsD protein (Fig. 5A) indicating that CDB3 ArsD is probably not a repressor. A sequence alignment between CDB3 ArsD and two biochemically characterised repressor ArsDs from *E. coli* R773 (Wu & Rosen, 1993) and *Acidiphilium multivorum* AIU 301 pKW301 (Suzuki et al., 1998) showed a distinct dissimilarity (Fig. 5B). Compared with the other two proteins, CDB3 ArsD does not possess the two C-terminal vicinal cysteine pairs, Cys^112^–Cys^113^ and Cys^119^–Cys^120^.

The regulatory function of ArsD in arsenic defence has been demonstrated in at least two species, *E. coli* and *A. multivorum* AIU 301 (Suzuki et al., 1998; Li, Chen & Rosen, 2001). However, the ArsD encoded by *Bacillus* sp. CDB3 *ars1* did not show any interactions with the operon promoter in our mobility assay. Li, Chen & Rosen (2001) predicted that the two vicinal cysteine pairs (Cys^112,113^ and Cys^119,120^) in the C-terminal region are involved in regulatory function in *E. coli* R773. Our result supports the proposition. Conservation of the three N-terminal cysteine residues (Cys^12^, Cys^13^ and Cys^18^), which were shown to be essential for the metallochaperone function of ArsD in *E. coli* R773 (Lin, Yang & Rosen, 2007), points to its function as a chaperone. The involvement of *arsD* in arsenic resistance of *Bacillus* sp. CDB3 *ars1* has been demonstrated (Bhat et al., 2011) and the role of its product as a chaperone waits to be confirmed.

## Conclusions

The eight-gene operon of *Bacillus* sp. CDB3 *ars1* has been revealed to express its genes in a more complicated way than other known *ars* operons. It represents another example of specific mRNA degradation in the transporter gene region and possibly the first case of transcription attenuation in *ars* operons. The possible coordination of the two transcripts and other factors involved in both the transcriptional and post-transcriptional processes warrant further investigation.

## Supplemental Information

10.7717/peerj.1230/supp-1 Data S1 Raw data for qPCR Figure S1. Raw data for qPCR results in Figure 2B
